# Mediating role of health literacy in relationship between frailty and health-related quality of life in community-dwelling older adults

**DOI:** 10.1371/journal.pone.0303164

**Published:** 2024-05-30

**Authors:** Hee-Sun Kim, Jinhee Kim, Ji-Ah Kim

**Affiliations:** 1 National Evidence-Based Collaborating Agency, Seoul, South Korea; 2 Department of Nursing, Chosun University Hospital, Gwangju, South Korea; Universitas Indonesia Fakultas Kedokteran, INDONESIA

## Abstract

**Purpose:**

The aim of this study was to investigate the mediating effects of health literacy on the relationship between frailty and health-related quality of life (HRQOL) among community-dwelling older adults.

**Methods:**

This study used the Korean Frailty and Aging Cohort Database (KFACD) for secondary data analysis. We selected data from 1,631 people without missing main variable values for analysis. Frailty was determined based on the modified Fried’s phenotype [MFP], and HRQOL was measured using the Korean version of the 5-level EuroQol questionnaire (EQ-5D-5L). Health literacy was assessed using the questions on the Behavioral Risk Factor Surveillance System (BRFSS) used by the U.S. Center for Disease Control and Prevention. To examine the mediating role of health literacy in the relationship between frailty and HRQOL, Baron & Kenny’s three-step mediating effect verification method was utilized.

**Results:**

The participants had a mean frailty score of 1.37±1.02, health literacy score of 8.56±2.59, and HRQOL score of 0.84±0.10. Frailty was negatively correlated with health literacy (r = -0.27, *p* < .001) and HRQOL (r = -0.32, *p* < .001), while health literacy was positively correlated with HRQOL (r = 0.34, *p* < .001). We observed that health literacy played a partial mediating role in the relationship between frailty and HRQOL.

**Conclusion:**

To increase older adults’ HRQOL, measures that directly prevent and manage frailty as well as interventions that target the enhancement of health literacy are needed.

## Introduction

Rapid population aging has sparked avid interest in healthy life years, healthy aging, and health-related quality of life (HRQOL) beyond simply extending the lifespan. Consequently, frailty—a condition that threatens the health of older adults—has garnered much attention as well [1]. Frailty refers to a state marked by increased vulnerability to stressors as a result of physiological system deterioration with aging [2]. In particular, frailty transforms the body into a physically vulnerable state to diseases, which may increase older adults’ dependence and lead to unfavorable outcomes, such as falls, hospitalization, institutionalization, and death, thereby impairing their HRQOL [2–4]. Thus, frailty should be aggressively prevented and managed in order to reduce the adverse impact of frailty on HRQOL.

The growing elderly population increases the prevalence of frailty, health care utilization, and healthcare costs and strains the national economy [5, 6]. To address these challenges, there is a growing focus on health literacy as a proactive approach to preventing functional decline among older adults [1, 7]. Health literacy refers to the capacity to comprehend and assess health information, which enables informed decisions for enhancing and maintaining well-being [8]. It assumes that engaging in health-related behaviors requires a solid understanding of one’s own health information [7]. Previous studies have shown that low health literacy among older adults is linked to unfavorable health behaviors, including poor use of healthcare [9], misunderstanding medical instructions [10], and poor medication adherence [11], and that these ultimately impact physical and mental health as well as HRQOL [12, 13]. Moreover, studies have shown an association between health literacy and frailty [14], with the potential for improvement of these factors through active learning programs [15]. However, research establishing the relationship among frailty, health literacy, and HRQOL is largely lacking.

A high level of HRQOL is an important indicator of successful aging [14]. The prevention and management of frailty are essential to developing strategies to enhance older adults’ HRQOL, which also requires measures involving health literacy. According to a previous study [15], health literacy is reported to be a predictor of frailty progression. However, this study identifies the mediating effect of health literacy on the relationship between frailty and HRQOL among community-dwelling older adults. The results of this study are expected to provide evidence for the possibility of utilizing health literacy in developing measures to improve HRQOL by preventing and managing frailty.

## Materials and methods

### Study design

This study was a secondary data analysis study using the Korean Frailty and Aging Cohort Database (KFACD).

### Data source

This study used the Korean Frailty and Aging Cohort Database (KFACD) for secondary data analysis. The KFACD, a database developed as part of the Korean Frailty and Aging Cohort Study (KFACS), was analyzed. The KFACS registered 3,011 community-dwelling older adults aged 70–84 y in 2016 and 2017, and a second survey for each batch was conducted 2 y later, in 2018 and 2019, respectively. The main contents of the survey included frailty, activities of daily living, types of morbidity, mental health, health care utilization, health literacy, HRQOL, health behaviors, cognitive function, and social function. In the present study, data from 1,631 participants without any missing data for the major study variables were analyzed. Data analysis was conducted from May 15 to June 30, 2023.

### Measurement

#### 1. Frailty

Frailty status was determined using the modified version of Fried’s frailty phenotype, which was adapted from data from the Cardiovascular Health Study and has been regularly tested in the Asia-Pacific region, including South Korea [3, 16]. Fried’s phenotypes consist of five criteria: weight loss, weakness, exhaustion, slowness, and low physical activity. Each criterion is assigned a value of 1 if the condition is present and 0 if it is not present [3, 17]. The total score is the sum of the scores for each criterion and ranges from 0–5. A higher score indicates a greater degree of frailty.

#### 2. Health-related quality of life (HRQOL)

HRQOL was measured using the Korean version of the 5-level EuroQol questionnaire (EQ-5D-5L) [18, 19]. EQ-5D-5L consisted of five domains (mobility, self-care, usual activities, pain/discomfort, and anxiety/depression) and was measured at five levels each (1 = no problems, 2 = slight problems, 3 = moderate problems, 4 = severe problems, and 5 = extreme problems). The utility score is calculated by assigning weights, which was presented in the previous study [18], according to the level of each domain. A utility score of 1.0 means perfect health, 0 indicates death, and the possible range of utility values is −0.066 to 1.

#### 3. Health literacy

Health literacy was assessed using three questions used in the Behavioral Risk Factor Surveillance System conducted by the U.S. Center for Disease Control and Prevention [20]. The questions were as follows: (a) “How difficult is it for you to get advice about health or medical topics if you need it?” (b) “How difficult is it for you to understand information that doctors, nurses, and other health professionals tell you?” and (c) “You can find written information about health on the internet, in newspapers and magazines, and brochures in the doctor’s office and clinic; in general, how difficult is it for you to understand written health information?” Each item in the system is rated on a four-point Likert scale from 1, “very difficult” to 4, “very easy,” and the total score is the sum of the scores for each item. The total score ranges from 3–12, and a higher score indicates higher health literacy. The reliability of the tool (Cronbach’s alpha) was .71 in a previous study [21] and .98 in this study.

### Ethical considerations

This study was approved by the Institutional Review Board of the NECA (IRB No. NECA IRB21-020-8). This study complied with study data disclosure and utilization regulations of the NECA.

### Data analysis

#### Data were analyzed using the SPSS statistical software, version 24 (IBM)

General characteristics were expressed in terms of frequencies and percentages. Means and standard deviations were calculated to determine the level of frailty, health literacy, and HRQOL. To analyze differences in the levels of frailty, health literacy, and HRQOL based on the respondent’s general characteristics, a t-test or one-way analysis of variance was utilized. Pearson’s correlation coefficient was used to examine the relationships among frailty, health literacy, and HRQOL.

To examine the mediating role of health literacy in the relationship between frailty and HRQOL, we utilized Baron and Kenny’s [22] three-step mediating effect verification method. According to Baron and Kenny [22], a mediating role can be identified via the following regression equations: Step 1: regressing the mediator on the independent variable; Step 2: regressing the dependent variable on the independent variable; Step 3: regressing the dependent variable on both the independent variable and mediator. In this case, the general characteristic variables for which statistically significant differences were identified in the dependent variable were adjusted. The following conditions must hold true to establish mediation from the results: i) the independent variable must affect the mediator in the first equation, ii) the independent variable must affect the dependent variable in the second equation, and iii) the mediator must affect the dependent variable in the third equation. In this case, the effect of the independent variable on the dependent variable must be lower in the third regression analysis compared to the second one. If the relationship between the independent variable and the dependent variable is not statistically significant in the third equation, a complete mediating effect is suggested. If the relationship is significant, a partial mediating effect is suggested. We tested the significance of the mediating effect using the Sobel test.

## Results

### Characteristics of participants

Among the participants, 60.2% were male. The most age group with the most respondents was 70–75 y (50.5%), followed by 76–80 y (34.2%) and 81–84 y (15.4%). The mean age was 75.84 (±3.86) y. About 73.0% had a spouse, and 54.6% conformed to a religion. The most common educational atainment was elementary school or lower (40.4%), followed by college or higher (22.9%) and high school (21.5%). In terms of economic activity, most were unemployed (75.1%), and most rated their health as healthy (72.7%). A total of 50.4% were non-smokers, and 42.9% were ex-smokers. The most common drinking frequency was non-drinking (30.7%), followed by less than once a month (29.1%) and two to four times a month (15.6%) (Table 1).

**Table 1:** Participants’ characteristics and frailty, health literacy, and health related quality of life (n = 1,631).

Variables	Categories	N (%)	Frailty			Health literacy			Health related quality of life		
			M±SD	F or t	*p*	M±SD	F or t	*p*	M±SD	F or t	*p*
**Total**		1,631 (100.0)	1.37±1.02			8.56±2.59			0.84±0.10		
**Gender**	Male	982 (60.2)	1.42±0.97	2.17	.030	9.14±2.40	11.48	< .001	0.86±0.07	8.99	< .001
	Female	649 (39.8)	1.30±1.10			7.68±2.61			0.82±0.12		
**Age**		75.84±3.86									
	70–75	823 (50.5)	1.13±0.87	71.31	< .001	9.02±2.46	36.56	< .001	0.86±0.08	17.99	< .001
	76–80	557 (34.2)	1.47±1.04			8.35±2.53			0.83±0.11		
	81–84	251 (15.4)	1.96±1.17			7.52±2.76			0.82±0.12		
**Spouse**	Partnered	1,190 (73.0)	1.35±0.99	1.49	.136	8.92±2.50	-9.45	< .001	0.86±0.09	-7.51	< .001
	Single	441 (27.0)	1.44±1.10			7.59±2.58			0.81±0.13		
**Religion**	Follow	890 (54.6)	1.36±1.01	0.60	.547	8.60±2.50	-0.65	.513	0.84±0.09	0.14	.889
	Do not follow	741 (45.4)	1.39±1.04			8.51±2.69			0.84±0.10		
**Educational level**	≤ Elementary	659 (40.4)	1.56±1.12	12.29	< .001	7.24±2.47	138.13	< .001	0.81±0.12	46.64	< .001
	Middle school	247 (15.1)	1.28±0.99			8.69±2.31			0.86±0.07		
	High School	351 (21.5)	1.28±0.96			9.37±2.24			0.86±0.07		
	≥ College	374 (22.9)	1.21±0.87			10.05±2.08			0.87±0.05		
**Economic activity**	In employment	406 (24.9)	1.37±0.97	0.01	.996	8.71±2.57	-1.32	.188	0.86±0.08	-3.34	< .001
	Unemployed	1,225 (75.1)	1.37±1.04			8.51±2.59			0.84±0.10		
**Self-rated health status**	Healthy	1,185 (72.7)	1.20±0.90	10.46	< .001	9.02±2.46	-12.17	< .001	0.86±0.07	-11.64	< .001
	Unhealthy	446 (27.3)	1.84±1.17			7.34±2.51			0.79±0.13		
**Smoking**	Non-smoker	822 (50.4)	1.32±1.07	4.49	.011	8.11±2.66	26.79	< .001	0.83±0.11	31.51	< .001
	Ex-smoker	699 (42.9)	1.40±0.96			9.05±2.39			0.86±0.07		
	Smoker	110 (6.7)	1.61±1.01			8.82±2.68			0.87±0.06		
**Drinking**	Non-drinking	501 (30.7)	1.54±1.10	6.29	< .001	8.21±2.60	7.25	< .001	0.83±0.11	9.69	< .001
	≤1 time/month	475 (29.1)	1.32±1.01			8.42±2.54			0.83±0.10		
	2–4 times/month	255 (15.6)	1.19±0.94			9.15±2.54			0.86±0.07		
	2–3 times/week	221 (13.5)	1.27±0.97			8.94±2.56			0.87±0.07		
	≥4 times/week	179 (11.0)	1.44±0.94			8.60±2.60			0.85±0.08		

M±SD = Mean±Standard deviation

### Frailty based on participants’ characteristics

The mean frailty score was 1.37±1.02, and frailty significantly differed according to gender (t = 2.17, *p* = .030), age (F = 71.31, *p* < .001), educational attainment (F = 12.29, *p* < .001), self-rated health status (t = 10.46, *p* < .001), smoking (F = 4.49, *p* = .011), and drinking (F = 6.29, *p* < .001). Frailty level was higher in males (1.42±0.97), those aged 81–84 y (1.96±1.17), with an elementary school education or lower (1.56±1.12), those who rated themselves as unhealthy (1.84±1.17), smokers (1.61±1.01), and non-drinkers (1.54±1.10) (Table 1).

### Health literacy based on participants’ characteristics

The mean health literacy score was 8.56±2.59, and it significantly differed according to gender(t = 11.48, *p* < .001), age (F = 36.56, *p* < .001), spouse (t = -9.45, *p* < .001), educational attainment (F = 138.13, *p* < .001), self-rated health status (t = -12.17, *p* < .001), smoking (F = 26.79, *p* < .001), and drinking (F = 7.25, *p* < .001). Health literacy levels were higher in males (9.14±2.40), those aged 70–75 years (9.02±2.46), those who had a spouse (8.92±2.50), with a college education or higher (10.05±2.08), those who rated themselves as healthy (9.02±2.46), ex-smokers (9.05±2.39), and those who drank two to four times a month (9.15±2.54) (Table 1).

### Health-related quality of life based on participants’ characteristics

The mean HRQOL score was 0.84±0.10, and it significantly differed according to gender (t = 8.99, *p* < .001), age (F = 17.99, *p* < .001), spouse (t = -7.51, *p* < .001), educational attainment (F = 46.64, *p* < .001), economic activity (t = -3.34, *p* < .001), self-rated health status (t = -11.64, *p* < .001), smoking (F = 31.51, *p* < .001), and drinking (F = 9.69, *p* < .001). HRQOL was higher in males (0.86±0.07), those aged 70–75 y (0.86±0.08), those who had a spouse (0.86±0.09), with a college education or higher (0.87±0.05), employed individuals (0.86±0.08), those who rated themselves as healthy (0.86±0.07), smokers (0.87±0.06), and those who drank two to three times a week (0.87±0.07) (Table 1).

### Correlations among frailty, health literacy, and health-related quality of life

Frailty and health literacy had a statistically significant, negative correlation (r = -0.27, *p* < .001). Frailty and HRQOL also had a statistically significant, negative correlation (r = -0.32, *p* < .001). Health literacy and HRQOL had a statistically significant, positive correlation (r = 0.34, *p* < .001) (Table 2).

**Table 2:** Correlation among frailty, health literacy, and health related quality of life (n = 1,631).

Variable	Frailty	Health literacy	Health related quality of life
	r(*p*)	r(*p*)	r(*p*)
Frailty	1		
Health literacy	-0.27 (< .001)	1	
Health related quality of life	-0.32 (< .001)	0.34 (< .001)	1

### Mediating effect of health literacy in relationship between frailty and health-related quality of life

A three-step regression analysis was used to examine the mediating effect of health literacy in the relationship between frailty and HRQOL [22] (Table 3).

**Table 3:** Mediating role of health literacy in the relationship between frailty and health related quality of life (n = 1,631).

Steps	Direction	β (*p*)	t	R^2^	Adj. R^2^	F (*p*)
Step 1	Frailty → Health literacy	-0.15 (< .001)	-6.43	.311	.308	91.58 (< .001)
Step 2	Frailty → Health related quality of life	-0.22 (< .001)	-9.41	.254	.249	55.06 (< .001)
Step 3	Frailty, Health literacy → Health related quality of life			.265	.260	53.12 (< .001)
	Health literacy → Health related quality of life	0.13 (< .001)	5.04			
	Frailty → Health related quality of life	-0.20 (< .001)	-8.56			

Gender, age, spouse, educational level, self-rated health status, smoking, and drinking were adjusted in step1.

Gender, age, spouse, educational level, economic activity, self-rated health status, smoking, and drinking were adjusted in step 2 and 3.

To check for multicollinearity of the independent variables, a basic assumption of regression analysis, we computed the Durbin-Watson statistic, tolerance, and variance inflation factor (VIF). The Durbin-Watson statistic was close to 2, at 1.95–1.97, confirming the absence of autocorrelation. Tolerance was above 0.1, at 0.34–0.94, and VIF was below 10, at 1.07–2.98, confirming the absence of multicollinearity. An analysis of residuals also confirmed the linearity and equal variance of the model.

The results of the three-step regression analysis are presented as follows: In Step 1, we observed a statistically significant negative impact of frailty on health literacy (β = -0.15, *p* < .001). In Step 2, we observed that frailty had a statistically significant negative impact on HRQOL (β = -0.22, *p* < .001). In Step 3, a model that considered frailty, as an independent variable, and health literacy, as the mediator, revealed that health literacy had a statistically significant positive impact on HRQOL (β = 0.13, *p* < .001). Additionally, the model indicated that the β value of frailty was equal to -0.20, which was lower than that from the second analysis (-0.22). Furthermore, this model highlighted the statistically significant impact of frailty on HRQOL (*p* < .001). Therefore, our results suggest that health literacy plays a partial mediating role in the relationship between frailty and HRQOL. The Sobel test confirmed that health literacy had a significant mediating effect between frailty and HRQOL (Z = -3.95, *p* < .001).

## Discussion

HRQOL is defined as the individual’s perceived health status, reflecting subjective feelings and satisfaction. A good HRQOL is the ultimate goal of all health interventions [23]. The aim of this study was to investigate the mediating effect of health literacy on the relationship between frailty and HRQOL among community-dwelling older adults in order to develop strategies to boost HRQOL in this population.

In our study, the mean frailty score was 1.37±1.02. While a direct comparison is difficult due to the current lack of studies using the same tool, a previous study in Japan [15] yielded a frailty level of 1.5±1.0 using the Japanese frailty indicator (25 items). This was a lower level of frailty compared to that of the participants of our study. In a study conducted in Taiwan [24], older adults residing in two rural counties were assessed using the Taiwanese version of the Tilburg Frailty Indicator (15 questions), and the mean score was 5.69±3.22. Considering that the cutoff value for frailty in this instrument was 5.5 [25], a higher prevalence of frailty was observed in these individuals compared to that of our study population. It is difficult to determine differences in frailty levels and identifying a concrete cause of such gaps in frailty levels across countries, and further research comparing the levels of frailty using nationally representative samples is essential. An expanding older adult population is anticipated to lead to an increase in the prevalence of frailty [5, 6]. Previous studies [26–28] have suggested exercise, nutritional management, monitoring for polypharmacy, cognitive interventions, fall management, and social management of frailty as some strategies to prevent and manage frailty. These measures should be developed and implemented in communities where older adults reside, and national projects that aid the implementation of these measures should be launched. In Korea, the National Health Plan 2030 (2021–2030) [29] includes plans to transition to a comprehensive healthcare service system to incorporate frailty management. Such efforts of the Korean government should focus on supporting the healthy aging of the Korean population.

The mean health literacy score among our participants was 8.56±2.59. While a direct comparison is difficult due to the current lack of studies using the same tool, a study conducted on adults aged 18 y and over [30] defined low health literacy as having difficulty in at least one of the three items using the same tool, and the prevalence of low health literacy was 15.3%. A study conducted on older adults [15] used the five-item questionnaire developed by Ishikawa et al. [31] (5-point Likert scale) and defined low health literacy as an average score of <3; with this cutoff, the prevalence of low health literacy was 23.2%. Advancing age impairs one’s health literacy [32]. However, health literacy is modifiable and can be improved through training and education [33]. A high level of health literacy brings about positive changes in attitude and behaviors, including the quality of diet, smoking cessation, and medical adherence; and drives a healthy lifestyle, such as increased involvement in physical activities and the enjoyment of diverse diets [34–36]. The World Health Organization stressed the need to recognize health literacy as a determinant of health and emphasized that countries should implement national-level policies and strategies in order to improve health literacy [37]. The Korean government should also devise national policies targeting the improvement of health literacy among older adults. Additionally, healthcare professionals in primary care settings who provide care to older adults at the frontline should be aware of the issues pertaining to health literacy, provide information in an easy-to-understand manner for older adults, and confirm that they have adequately understood the given information.

The level of HRQOL among healthy people was reported as 0.96±0.08 [38], and that among people with chronic conditions was reported as 0.64±0.31 [39]. In our study conducted on community-dwelling older adults aged 70–84 y, the mean HRQOL score was 0.84±0.10, lower than that among healthy people but higher than that among people with chronic conditions. The older adult population is projected to continue to grow as a result of advances in healthcare technology and standards of living [40]. A high level of HRQOL is an important indicator of successful aging [14], and it is the ultimate goal of all health interventions [23]. There is a need for national-level policies and projects to boost HRQOL among older adults.

The findings of this study showed a negative relationship between frailty and HRQOL, which are consistent with previous findings [2–4, 24]. To ensure an adequate HRQOL among community-dwelling older adults, frailty assessment should become a routine practice in primary care settings in addition to frailty management projects, such that older adults at risk for impaired HRQOL could be identified early. To this end, simple and convenient frailty diagnostic instruments should be developed and disseminated.

We also found a negative relationship between frailty and health literacy. This is in the same context as previous findings, showing that health literacy declines with advancing age [32]. Health literacy deteriorates in older adulthood, when individuals experience a decline in their cognitive processes [41]. Considering that the growing older adult population is linked to an increased prevalence of frailty, health literacy should be improved through aggressive preventive and management measures for frailty.

We observed a positive relationship between health literacy and HRQOL, which is consistent with previous findings [12, 13]. Health literacy facilitates a healthy lifestyle, thereby having a positive impact on HRQOL [34–36]. To ensure adequate HRQOL among older adults, more attention and necessary measures need to be implemented at the national and local levels to improve health literacy.

In our study, we found that health literacy partially mediates the relationship between frailty and HRQOL. These results suggest that although HRQOL declines as a result of frailty among community-dwelling older adults, it can be increased by improving their health literacy. Thus, measures that directly prevent and manage frailty as well as interventions that target the enhancement of health literacy are needed to improve older adults’ HRQOL.

This study had the following limitations. First, we only included participants registered in the KFACD in our analysis, so the findings cannot be generalized to the entire older adult Korean population. The results of this study should be verified using data representative of the older adult Korean population. Second, the data analyzed in this study were obtained via self-report questionnaires, and participants who had difficulty responding to the questionnaire were excluded, increasing the risk of selection bias. The results should be interpreted in consideration of this limitation. Third, due to limited data, this study was unable to include mental-health-related variables in the analysis that could affect the main variables of this study. However, the KFACS excluded participants with cognitive impairment during selection. These issues should be considered when interpreting the study results. Fourth, Fried et al. [3] classified the frailty levels, as presented in a modified version of Fried’s frailty phenotype, as follows: if it corresponds to 0 out of 5 items, it is robust; if it corresponds to 1 to 2 items, it is pre-frail, and if it corresponds to 3 or more items, it is frail. However, as this study analyzed frailty, assuming it was a continuous variable ranging from 0 to 5, this aspect should be considered when interpreting the study results.

## Supporting information

S1 Data (XLS)
